# Sensor Array and SMOTE-Based Algorithms for Volatile-Fingerprint Classification of Pesticide-Treated Soil with a Novel Chamber

**DOI:** 10.3390/s26154763

**Published:** 2026-07-27

**Authors:** Shixiao Yu, Jiayi Li, Hang Yu, Zhiqiong Wang, Yingkui Xiao, Jingchun Wang, Zhiyong Chang

**Affiliations:** 1 Key Laboratory of Bionic Engineering, Ministry of Education, Jilin University, Changchun 130022, China; yusx25@mails.jlu.edu.cn (S.Y.); lijiayi@jlu.edu.cn (J.L.); xiaokuer0623@gmail.com (H.Y.); xiaoyk@jlu.edu.cn (Y.X.); 2 School of Mechanical and Aerospace Engineering, Jilin University, Changchun 130022, China; 3 School of Engineering and Technology, China University of Geosciences, Beijing 100083, China

**Keywords:** electronic nose, sensor array, volatile-fingerprint classification, pesticide-treated soil, gas-sensing chamber, Synthetic Minority Over-sampling Technique, Geometric SMOTE, imbalanced data

## Abstract

**Highlights:**

**What are the main findings?**

- Designed a mirror-symmetric conformal gas-sensing chamber for a 26-sensor metal oxide semiconductor (MOS) array to improve gas-flow uniformity and signal repeatability.
- Evaluated the Synthetic Minority Over-sampling Technique (SMOTE) and Geometric SMOTE (G-SMOTE) using training-fold-only oversampling to improve minority-class recognition in controlled cross-validation.

**What are the implications of the main findings?**

- Shows that class-specific metrics should be considered together with average accuracy when evaluating imbalanced pesticide-residue classification.
- Provides an efficient framework for monitoring minority pesticide classes in practical, complex environments, aiding food safety and environmental assessment.

**Abstract:**

Soil pesticide-residue screening is important for ecological protection, food safety, and public health. However, conventional chromatographic and spectroscopic methods often require complex sample pretreatment, expensive instruments, trained operators, and long analysis times, which limits their use for rapid and large-scale screening. In this study, an electronic-nose system was developed for volatile-fingerprint classification of pesticide-treated loess soil. Six commercial pesticide formulations from three chemical categories were evaluated: deltamethrin and cyfluthrin as pyrethroids, glyphosate and chlorpyrifos as organophosphorus pesticides, and zineb and mancozeb as organosulfur pesticides. These compounds were selected to represent commonly used pesticides with different chemical structures and volatile profiles. A mirror-symmetric gas-sensing chamber was designed for a 26-sensor metal oxide semiconductor (MOS) array to improve gas-flow uniformity and response repeatability. A total of 960 pesticide-treated electronic-nose response curves were collected from four soil depths. Eight feature extraction methods and four classifiers were compared. The Synthetic Minority Over-sampling Technique (SMOTE) and Geometric SMOTE (G-SMOTE) were then evaluated using training-fold-only oversampling to reduce data-leakage risk in imbalanced classification. The results showed that k-nearest neighbors (KNN) combined with direct or transform-based features provided strong classification performance under controlled laboratory conditions. For minority-class recognition, SMOTE showed more consistent improvement than G-SMOTE in the tested pesticide–depth–feature combinations, although the effect depended on feature representation and pesticide class. These findings indicate that the proposed chamber/sensor-array/SMOTE framework is feasible for rapid volatile-fingerprint classification of pesticide-treated soil, but larger independent field datasets and quantitative chemical validation are still required before general deployment.

## 1. Introduction

Soil is an indispensable foundation for agricultural production, supporting the growth of vital food and economic crops such as wheat, corn, soybeans, and potatoes. However, many agricultural regions face challenges posed by specific climatic and soil conditions, which often lead to inherently low levels of natural organic matter and weak nutrient retention capacity in the soil [1,2]. To mitigate pest threats and ensure necessary crop yields, the application of pesticides remains unavoidable in agricultural practice. Studies indicate that over seventy percent of applied pesticides do not reach their target organisms but instead persist and accumulate in the soil environment [3,4]. Under the influence of soil dynamics and seasonal rainfall, these residual pesticides are highly susceptible to migration through water, leading to leaching or runoff [5].

Conventional pesticide-residue analysis, including chromatographic and spectroscopic methods [6,7], remains accurate and sensitive but is not always suitable for rapid and large-scale soil screening. Chromatographic methods such as GC-MS/MS, LC-MS/MS, and UPLC-MS/MS usually require solvent extraction, cleanup, matrix-matched calibration, expensive instruments, and trained operators. Spectroscopic methods are faster and less destructive, but their performance can be affected by soil-matrix heterogeneity, moisture variation, spectral preprocessing, and model transfer. Therefore, a rapid, low-cost, and sensor-based screening approach is still needed for preliminary classification of pesticide-treated soil samples. Nazarloo et al. combined near-infrared spectroscopy and gas chromatography and compared partial least squares regression (PLSR) and artificial neural network (ANN) models under ten spectral preprocessing methods for nondestructive pesticide-residue prediction in tomatoes [8]. Lin employed ultra-performance liquid chromatography coupled with tandem triple quadrupole mass spectrometry (UPLC-MS/MS) to determine 30 banned pesticide residues using five different methodologies, with corresponding optimizations of pretreatment methods, chromatographic separation, and mass spectrometric conditions [9]. Vidal et al. categorized pesticides into polar and non-polar groups, applying gas chromatography for non-polar pesticides and ultra-performance liquid chromatography (UPLC) for polar ones [10]. Many researchers continue to explore novel and efficient rapid detection methods, among which sensor-based detection has attracted widespread attention due to its advantages of high efficiency, sensitivity, ease of integration, and low power consumption [11,12,13].

Despite the advantages of e-nose systems, two issues remain insufficiently addressed in soil pesticide volatile-fingerprint classification. First, soil matrices contain complex background volatile compounds, which may interfere with pesticide-specific sensor responses. Second, pesticide residues in real agricultural environments are often imbalanced across classes because some pesticides are frequently used whereas emerging or less commonly used pesticides occur at much lower levels. This imbalance may cause conventional classifiers to favor majority classes and fail to identify minority pesticide residues. These two challenges motivated the development of a hardware–algorithm framework for improving gas-delivery consistency and minority-class recognition in e-nose-based soil pesticide detection.

Accordingly, the main contributions of this study are threefold: first, a mirror-symmetric conformal gas-sensing chamber was designed and optimized to improve gas delivery uniformity across a 26-sensor MOS array; second, eight feature extraction methods and four classifiers were compared to determine suitable recognition configurations; third, SMOTE and G-SMOTE were evaluated using training-fold-only oversampling to improve minority-class pesticide recognition while reducing the risk of data leakage.

The objective of this study was not to quantify pesticide residues, but to evaluate laboratory-scale volatile-fingerprint classification of pesticide-treated loess soil under imbalanced sampling conditions.

## 2. Materials and Methods

### 2.1. Soil and Pesticides

#### 2.1.1. GC-MS Analysis of Pesticide Volatile Compounds

Volatile compounds from the pesticide formulations were analyzed using an Agilent 5975 gas chromatography–mass spectrometry system (Agilent Technologies, Santa Clara, CA, USA). Solid-phase microextraction was performed using a 65 μm PDMS/DVB fiber purchased from Supelco (Bellefonte, PA, USA). Before extraction, each pesticide sample was placed in a sealed headspace vial and equilibrated at 60 °C for 30 min. The PDMS/DVB fiber was then exposed to the headspace at the same temperature for 20 min to collect volatile organic compounds. After extraction, the fiber was immediately inserted into the GC-MS injection port and thermally desorbed at 250 °C for 1 min.

The volatile compounds were separated on an HP-5MS fused-silica capillary column (Agilent Technologies, Santa Clara, CA, USA) with a length of 30 m, an inner diameter of 0.25 mm, and a film thickness of 0.25 μm. Helium was used as the carrier gas at a flow rate of 1 mL/min. The oven temperature program was as follows: the initial temperature was maintained at 40 °C for 3 min, increased to 280 °C at a rate of 10 °C/min, and then held at 280 °C for 15 min. The mass spectrometer was operated in electron ionization mode at 70 eV. The ion source temperature was 230 °C, the quadrupole temperature was 150 °C, and the scan range was 20–550 amu. Compounds were identified by matching the obtained mass spectra with the NIST 2008 mass spectral library (National Institute of Standards and Technology, Gaithersburg, MD, USA).

It should be noted that GC-MS was used here to characterize the volatile profiles of the pesticide formulations and to support sensor-array selection; it was not used to quantify pesticide-residue concentrations in the post-leaching soil layers.

#### 2.1.2. Soil Sample Preparation

The loess soil samples were collected from the Weihai Institute of Bionics of Jilin University (located at latitude 36°41′–37°35′ N, longitude 121°11′–122°42′ E). This study selected three different types of pesticides—pyrethroids, organophosphorus, and organosulfur compounds—with two specific pesticides chosen from each category for detection: deltamethrin and cyfluthrin [14], glyphosate [15], chlorpyrifos [16], and zineb and mancozeb [17]. The six pesticide working solutions were prepared from commercial formulations according to their product instructions, and their active ingredient contents, including 2.8% deltamethrin, 5.7% cyfluthrin, 41% glyphosate, 5% chlorpyrifos, 80% zineb, and 80% mancozeb, are listed in Table S1. The leaching experiment was conducted according to GB/T 31270.5-2014, “Test Guidelines on Environmental Safety Assessment for Chemical Pesticides—Part 5: Soil Leaching Test” [18]. Briefly, loess soil was packed and compacted into glass columns to form a 40 cm soil layer. Before pesticide application, distilled water was slowly added to pre-wet all soil columns and simulate natural moist agricultural soil conditions; however, a fixed gravimetric moisture-content value was not imposed. Six pesticide solutions were then uniformly added to the corresponding leaching columns. A blank control was prepared using distilled water without pesticide addition. To reduce pesticide photodegradation, all treatments were conducted in a ventilated fume hood under dark conditions. Subsequently, 800 mL of 0.01 mol/L CaCl_2_ solution was used for leaching each soil column to simulate rainfall-driven pesticide migration.

After leaching, each soil column was divided into four depth intervals, namely 0–10 cm, 10–20 cm, 20–30 cm, and 30–40 cm, while maintaining the original soil structure as much as possible. For each depth interval, 10 g of soil was collected and sealed in a beaker for subsequent volatile gas analysis using the electronic nose system. The blank control samples were divided and analyzed using the same procedure as the pesticide-treated samples.

Because the purpose of this study was to evaluate volatile-fingerprint classification and minority-class recognition rather than to establish a quantitative residue-concentration model, the actual post-leaching pesticide concentrations in different soil layers were not chemically quantified.

The experimental design included six pesticide treatments and one blank-control treatment, and each treatment was divided into four soil-depth intervals. For each pesticide-depth combination, 40 repeated electronic-nose measurements were obtained, and each measurement generated one 60 s response curve for feature extraction and classification analysis. These repeated measurements were treated as technical replicates for model development.

### 2.2. Design of the Gas Sensing Chamber for the Electronic Nose

The design of the chamber is primarily based on the structural and functional characteristics of the mammalian olfactory system. Its core components include the electronic nasal chamber structure and a sensor array. Unlike existing electronic noses used for pesticide detection, the novel chamber structure is designed with the goal of achieving uniform detection across a 26-sensor array, incorporating bionic principles to create an integrated structure combining a conformal circle and a flow-guiding chamber. The 26 MOS sensors were arranged in a mirror-symmetric layout on the upper and lower sides of the chamber. This configuration was designed to expose all sensors to the incoming volatile gas flow as synchronously and uniformly as possible. The conformal circular region increased the gas–sensor contact area, whereas the flow-guiding chamber helped distribute the gas flow across the sensor surfaces and reduce local stagnation or uneven concentration zones.

#### 2.2.1. Chamber Parameter Optimization Experiment

To determine the optimal geometric parameters of the chamber, single-factor and multi-factor orthogonal experiments were designed with the primary objective of maximizing and ensuring uniformity of flow velocity on the sensor surfaces. The single-factor experiment selected four key factors influencing airflow, each with five levels, while keeping other parameters fixed at baseline values to investigate the impact of individual factors on the sensor surface flow velocity, as shown in Table 1. Based on the results of the single-factor experiment, a four-factor, five-level orthogonal experiment was designed for the multi-factor study, comprising a total of 25 test groups that covered all parameter combinations. Accordingly, both the mean sensor-surface velocity and the inter-position velocity variation were used as optimization criteria.

#### 2.2.2. Computational Fluid Dynamics (CFD) Simulation

Computational fluid dynamics (CFD) simulation was performed using Fluent (version 2022 R2, Ansys Inc., Canonsburg, PA, USA) software to evaluate the flow-field distribution inside the gas-sensing chamber. The 3D chamber model (in x_t format), created with Creo (version 8.0, PTC Inc., Boston, MA, USA), was imported into SpaceClaim (Ansys 2022 R2), where duplicate edges and faces were removed. Cylinders with a height of 7 mm and a diameter of 8 mm were extruded on the upper and lower sides of the chamber to simulate the actual sensors. These were set as solid materials to form a closed fluid domain.

In Fluent Meshing (Ansys 2022 R2), non-fluid structures such as the chamber shell and sensors were set as “dead” (no fluid flow), retaining only the gas flow region to reduce computational load. Due to the chamber’s symmetrical structure, only the surfaces of the 13 sensors on the upper side were selected as the analysis plane. The flow velocity distribution in this region was calculated based on the Navier–Stokes equations to avoid redundant computations.

A tetrahedral mesh was selected for discretization. The orthogonal quality of the surface mesh mostly ranged between 0.8 and 1.0, while the orthogonal quality of the volume mesh was concentrated around 0.9, with a minimum value of approximately 0.2. Based on the chamber dimensions, the total number of mesh elements was between 500,000 and 800,000. The fluid medium was set as air at room temperature (25 °C) with a density of 1.225 kg/m^3^ and a viscosity of 1.789 × 10^−5^ Pa·s. The wall conditions were defined with the chamber walls and sensor surfaces set as no-slip walls. The SIMPLE algorithm was employed for solving, with a convergence residual set to 10^−6^ to ensure stable results.

### 2.3. Selection of the Sensor Array

Metal oxide semiconductor (MOS) gas sensors are a common type of gas detector widely used for monitoring the concentration of harmful gases and volatile organic compounds (VOCs) in the air [19]. Their working principle is based on the interaction between the metal oxide semiconductor and the target gas molecules. These sensors consist of a thin film of metal oxide, such as tin oxide (${SnO}_{2}$) or zinc oxide (ZnO), deposited on a ceramic or silicon substrate [20]. When the target gas is present, gas molecules adsorb onto the surface of the metal oxide semiconductor, resulting in a change in the semiconductor’s electrical resistance, typically manifesting as a decrease in resistance. By measuring this change in resistance, the sensor can determine the concentration level of the target gas, offering high sensitivity and rapid response [21].

MOS gas sensors are the most commonly used sensor type in electronic nose (e-nose) systems due to their high sensitivity, albeit poor selectivity, making them well suited for e-nose applications [22]. Furthermore, considering the primary components of the target gases to be detected and the overall system cost, this system selected an array of 26 MOS sensors from Figaro (Mino, Osaka, Japan) and Winsen (Zhengzhou, China). The selected MOS sensors show cross-sensitivity to the major volatile compound groups identified by GC-MS, including alcohols, aromatic hydrocarbons, halogenated hydrocarbons, aldehydes, ketones, ethers, sulfur-containing gases, and general VOCs. Although individual MOS sensors have limited selectivity, their combined response patterns provide useful multidimensional information for pesticide classification. The specific models of the selected sensors and their target gases, sensitivities, and resistance change rates are primarily listed in Table 2.

### 2.4. Data Acquisition and Processing Pipeline

Before each measurement, the electronic nose system was preheated for 1 h to stabilize the MOS sensor baseline. The prepared soil sample was placed in a beaker sealed with a 3D-printed sampling lid. The headspace gas generated from the soil sample was pumped into the gas-sensing chamber at a flow rate of 300 mL/min. Sensor signals were recorded at a sampling frequency of 100 Hz for 60 s. After each measurement, dry air was introduced into the gas path to purge the chamber and restore the sensor signals to baseline before the next test. All pesticide-treated and blank-control samples were measured using the same sampling time, gas flow rate, purge procedure, and laboratory conditions to minimize environmental variation.

**Figure 1:**
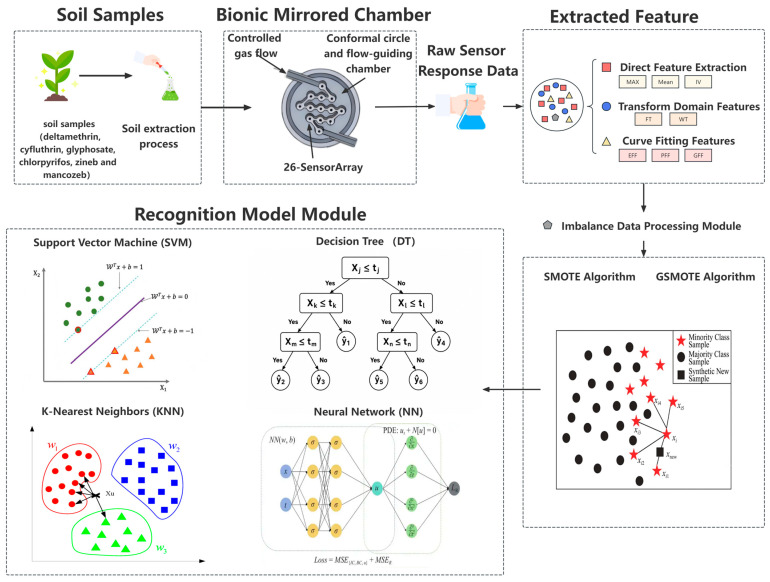
Data acquisition and processing workflow diagram.

The raw electronic-nose data consisted of time-dependent response curves from the 26-sensor MOS array. Before feature extraction, baseline correction was performed using the initial stable response of each sensor, and the corrected response curves were used to construct numerical features. Figure 1 summarizes the overall workflow from soil sampling and chamber detection to feature extraction, training-fold oversampling, model training, and evaluation. Detailed preprocessing and validation procedures are described in Section 2.4.3.

Potential interference from soil background volatiles was partially controlled by including distilled-water blank columns and by applying the same pre-wetting, leaching, sampling, and purge procedures to all treatments. However, moisture variation, pesticide co-formulants, and other soil VOC interferences were not independently varied in controlled interference experiments. Therefore, interference tolerance was not systematically validated and is discussed as a limitation.

#### 2.4.1. Feature Extraction

Feature extraction refers to the process of deriving salient information from raw data to facilitate enhanced representation, analysis, and comprehension [23]. The objective of feature extraction is to distill task-relevant information through the selection, transformation, or construction of features, while simultaneously minimizing the influence of extraneous data [24,25].

To evaluate the efficacy of diverse feature extraction methodologies, this study conducted a comparative analysis of eight distinct approaches. Three features were extracted directly from the raw response curves: maximum value (MAX), mean value (Mean), and integral value (IV). The mathematical formulations for these features are given by Equations (1)–(3):

(1) $$\mathrm{MAX}={\mathrm{MAXx}}_{t}$$

(2) $$\mathrm{Mean}=\frac{\sum_{i=1}^{n}x_{i}}{n}$$

(3) $$\mathrm{IV}=\int_{a}^{b}x(t)\mathrm{dt}$$

where $x_{t}$ represents the sensor response value at time t, and n denotes the response duration of the sensor.

Exponential function fitting (EFF), polynomial function fitting (PFF), and Gaussian function fitting (GFF) are curve-fitting-based methods for modeling the sensor response curves. Their feature extraction functions are defined by Equations (4)–(6):

(4) $$f\left(t\right)=a_{1}e^{b_{1}x_{t}}+a_{2}e^{b_{2}x_{t}}+\ldots +a_{1}e^{b_{n}x_{t}}$$

(5) $$f\left(t\right)=p_{0}{x_{t}}^{n}+p_{1}{x_{t}}^{n-1}+\ldots +p_{n}$$

(6) $$f\left(t\right)=\frac{1}{\sigma \sqrt{2\pi }}e^{\frac{1}{-2}{\left(\frac{t-\mu }{\sigma }\right)}^{2}}$$

where $f\left(t\right)$ denotes the probability density function, $x_{t}$ represents the sensor response value at time t, μ is the mean value indicating the central tendency of the data distribution, and σ is the standard deviation quantifying the dispersion of the data distribution.

Fourier transform (FT) and wavelet transform (WT) are fundamental signal processing techniques commonly employed in electronic nose signal analysis. Their feature extraction functions are defined by Equations (7) and (8):

(7) $$F\left(w\right)=\;F\left[f\left(t\right)\right]=\;\int_{-\infty }^{\infty }f\left(t\right)e^{-\mathrm{iwt}}\mathrm{dt}$$

(8) $$\mathrm{WT}\left(a\right.,\left.\tau \right)=\frac{1}{\sqrt{a}}\int_{-\infty }^{\infty }f\left(t\right)\times \varphi \left(\frac{t-\tau }{a}\right)\mathrm{dt}$$

where $f\left(t\right)$ denotes the sensor response value.

#### 2.4.2. Recognition Algorithms for Imbalanced Data

Compared to algorithms applied directly to balanced datasets, the core advantage of recognition algorithms for imbalanced datasets lies in their targeted approach to resolving data skew, thereby enabling more reliable decision-making in real-world scenarios [26]. This advantage manifests across multiple dimensions: Primarily, by adjusting data distribution, they actively direct the model’s attention towards minority class samples, significantly enhancing the capability to detect these underrepresented instances [27]. Secondarily, the strategic generation of synthetic samples facilitates the repositioning or refinement of decision boundaries. This process secures more equitable classification regions proximate to minority class clusters [28].

The SMOTE (Synthetic Minority Over-sampling Technique) algorithm operates on the minority class by first randomly selecting a sample point as the centroid. It then identifies k-nearest neighbors of this centroid as auxiliary points. Subsequently, for each auxiliary point, SMOTE generates n synthetic samples through linear interpolation along the line segment connecting the centroid to a randomly chosen auxiliary point [29].

In contrast, the G-SMOTE (Geometric SMOTE) algorithm diverges from this approach. Rather than being confined to linear interpolation between two points, G-SMOTE defines a geometric region centered on the seed sample. Within this bounded region, it controllably synthesizes new samples [30].

#### 2.4.3. Development and Evaluation of Recognition Models

Pattern recognition algorithms constitute a category of methods designed to identify and classify patterns within data, operating on the foundation of extracted features [31]. The efficacy of different feature extraction methods exhibits notable variation depending on data characteristics; consequently, comparative analysis of multiple feature extraction approaches facilitates the identification of optimal feature sets within specific detection contexts. Widely employed pattern recognition methodologies include:

Support Vector Machine (SVM): A prevalent supervised learning algorithm primarily utilized for classification and regression analysis. SVM demonstrates robust performance in solving both linear and nonlinear problems and excels at handling data in high-dimensional spaces [32].

Decision Tree: A widely used supervised learning algorithm for classification and regression tasks. It emulates human decision-making processes through a tree-like structure for data analysis and prediction. A decision tree comprises a root node, internal nodes, and leaf nodes. The root node signifies the initial feature for decision-making, internal nodes represent feature-based judgments, and leaf nodes denote the final classification outcome or numerical prediction [33].

K-Nearest Neighbors (KNN): A straightforward and efficient supervised learning method based on instance learning. It employs similarity measures to classify new samples or perform regression prediction. By identifying the k closest samples in the feature space to the target sample within the training set, KNN determines the new sample’s class through majority voting or its value through averaging [34].

Neural Network: A computational model inspired by the human nervous system, composed of numerous interconnected nodes (neurons). These nodes are organized in a hierarchical architecture, typically consisting of an input layer, one or more hidden layers, and an output layer. Information is processed and data features are learned through the adjustment of weights and biases during training [35].

The complete data-processing workflow consisted of baseline correction, feature extraction, data standardization, dataset splitting, oversampling of the training data, classifier training, and model evaluation. Raw response curves from the 26-sensor array were first converted into numerical features. The extracted features were then standardized using parameters estimated from the training data only. The learned standardization parameters were subsequently applied to the corresponding validation data.

Model performance was evaluated using ten-fold cross-validation. In each validation round, the dataset was divided into ten folds, with one fold used as the validation set and the remaining nine folds used for model training. For the imbalanced-data experiments, SMOTE and G-SMOTE oversampling were applied only to the training folds after the cross-validation split. The validation fold was kept unchanged and contained only original, non-synthetic samples. This procedure was used to avoid data leakage and to ensure that model performance was evaluated on independent experimental samples rather than synthetic data.

Because the study focuses on minority-class recognition, the recognition rate/recall of each minority pesticide class was reported in addition to average accuracy. This allowed the effect of oversampling on underrepresented classes to be evaluated directly rather than relying only on overall average accuracy.

The reported cross-validation results represent the mean performance across the ten validation folds. Repeated random cross-validation with different fold partitions and random seeds was not performed; therefore, variability caused by fold assignment and stochastic SMOTE/G-SMOTE sample generation was not fully quantified.

## 3. Results

### 3.1. GC-MS Profiles of Pesticide Volatile Compounds

The GC-MS results are summarized in Table 3 according to chemical classes. To improve transparency and reproducibility, a compound-level list is provided in Table S2, including pesticide name, CAS number, compound name, molecular formula and chemical class. The compound names, formulas, and chemical classes were identified by matching the mass spectra with the NIST 2008 library.

The GC-MS results showed distinct volatile organic compound profiles among the six pesticides. Glyphosate was dominated by alcohols and alkanes, with minor aromatic hydrocarbons, ethers, and esters. Chlorpyrifos, cyfluthrin, and deltamethrin were dominated by aromatic hydrocarbons, whereas mancozeb and zineb contained relatively higher proportions of alkanes, alcohols, and aromatic compounds. These compositional differences provide a chemical basis for interpreting the class-dependent e-nose responses observed in the subsequent classification analysis.

### 3.2. CFD Simulation Results

Based on the single-factor experiments and corresponding CFD simulations, the specific effects of various factors on the sensor surface flow velocity were obtained. A linear regression model was subsequently established with the conformal circle diameter (L), flow guide cavity thickness (R), inlet flow rate (Q), and transition zone width (P) as independent variables, and the sensor surface flow velocity (V) as the dependent variable. The resulting regression equation is:

V = 0.258 − 0.007L − 0.003R + 0.135Q − 0.011P(9)

**Figure 2:**
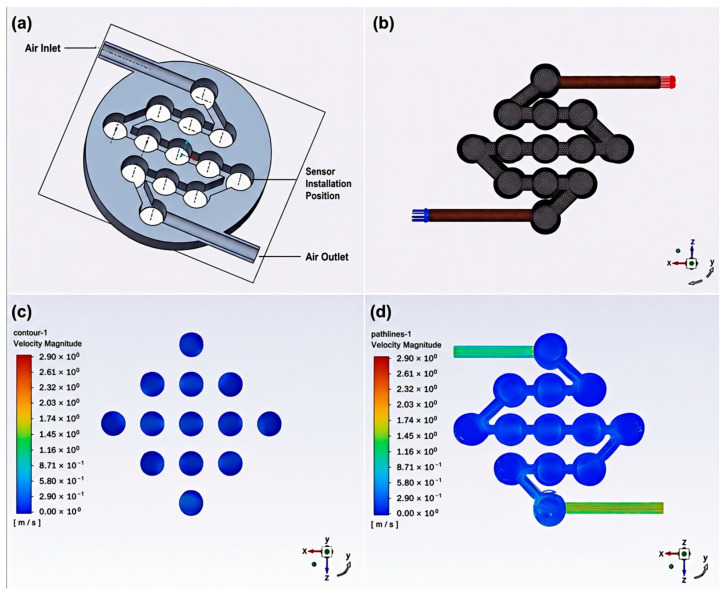
Gas detection chamber: (**a**) schematic diagram of the electronic nasal chamber design; (**b**) computational fluid domain model of the chamber; (**c**) contour plot of flow velocity on the sensor surface; (**d**) pathline diagram of gas flow velocity.

To evaluate the gas-flow distribution inside the chamber, the structural design and computational fluid dynamics simulation results of the gas detection chamber are presented in Figure 2.

The model exhibited a goodness-of-fit of R^2^ = 0.798. Significance testing results were as follows: F = 19.709, *p* = 0.000 < 0.05. The variance inflation factor (VIF) for multicollinearity and autocorrelation was below 5, and the Durbin–Watson (D-W) value was approximately 1.834, confirming the reliability of the model.

Considering the dual requirements of maximizing flow velocity and minimizing chamber size, the optimal parameter combination was identified as: conformal circle diameter: 9 mm; flow guide cavity thickness: 24 mm; inlet flow rate: 1.9 L/min; transition zone width: 4 mm.

### 3.3. Optimal Algorithmic Configuration

To evaluate classification performance, accuracy, precision, recall, F1-score, and multiclass ROC-AUC were considered. Table 4 reports average classification accuracy. Accuracy was calculated as the proportion of correctly classified samples among all validation samples. Precision, recall, and F1-score were used to further evaluate class-wise recognition performance, particularly for minority pesticide classes. For multiclass ROC-AUC, a one-vs-rest strategy was used. For each pesticide class, the target class was treated as the positive class and all remaining classes were treated as negative. The macro-AUC was then calculated as the unweighted mean of the class-wise AUC values.

(10) $$\mathrm{Accuracy}=\frac{\mathrm{TP}+\mathrm{TN}}{\mathrm{TP}+\mathrm{TN}+\mathrm{FN}+\mathrm{FP}}$$

Precision, recall, and F1-score were calculated as:

(11) $$\mathrm{Precision}\;=\;\frac{\mathrm{TP}}{\mathrm{TP}+\mathrm{FP}}$$

(12) $$\mathrm{Recall}=\frac{\mathrm{TP}}{\mathrm{TP}+\mathrm{FN}}$$

(13) $$F1\text{-}\mathrm{score}=2\times \frac{Precision\times Recall}{\left(Precision+Recall\right)}$$

As shown in Table 4, high apparent classification accuracies were obtained for several classifier–feature combinations under controlled laboratory cross-validation. In particular, KNN combined with MAX, Mean, PFF, IV, WT, and FT features produced accuracies close to or equal to 100% in some depth groups. However, its performance decreased markedly when EFF and GFF features were used. This suggests that, in this dataset, KNN performance was strongly influenced by the separability of the extracted feature space. Curve-fitting features may not have preserved sufficient class-discriminative information for all pesticide-depth combinations, whereas direct and transform-based features produced more separable response patterns.

The interpretation of these high-accuracy results and their generalization limitations are discussed in Section 4.

## 4. Discussion

Compared with the near-infrared approach of Nazarloo et al. [8], which used gas-chromatographic reference values and regression models to estimate pesticide residues, and the UPLC-MS/MS method of Lin et al. [9], which provided compound-specific quantitative analysis, the present electronic-nose approach offers rapid headspace-fingerprint acquisition but does not provide residue concentrations or analyte-specific confirmation. Likewise, the GC/QqQ-MS/MS and UPLC/QqQ-MS/MS workflow reported by Vidal et al. [10] illustrates the role of confirmatory chromatography for broad pesticide panels in soil. Therefore, the present system should be positioned as a preliminary classification or screening tool rather than a replacement for validated chemical analysis.

Unlike these quantitative or confirmatory methods, the main contribution of the present study lies in integrating a mirror-symmetric gas-sensing chamber, a 26-sensor metal oxide semiconductor array, and training-fold-only SMOTE/G-SMOTE oversampling for controlled-laboratory volatile-fingerprint classification. This integration provides a rapid classification framework, but quantitative chemical validation and independent field testing remain necessary before practical deployment.

### 4.1. Impact of Sample Reduction and Imbalance on Classification Performance

**Figure 3:**
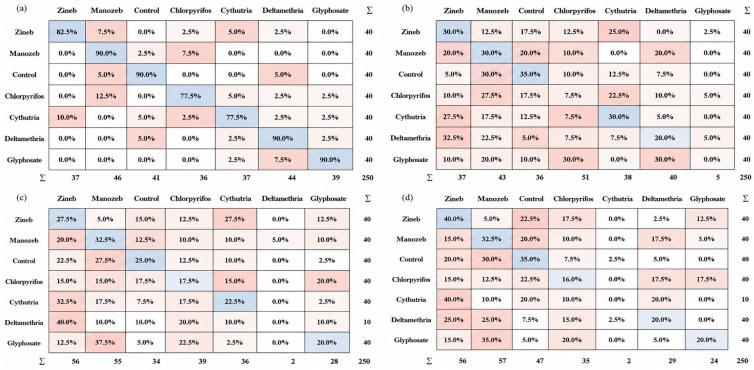
Comparative confusion matrices under balanced data conditions with a sample size of 10 per class: (**a**) confusion matrix for balanced data; (**b**) confusion matrix with glyphosate sample size reduced to 10; (**c**) confusion matrix with deltamethrin sample size reduced to 10; (**d**) confusion matrix with cyfluthrin sample size reduced to 10. Blue cells on the main diagonal represent correctly classified samples, whereas red cells in the off-diagonal positions represent misclassified samples. A darker color indicates a higher classification percentage.

To investigate the impact of imbalanced data on electronic nose (e-nose) detection results, we selected the dataset acquired at a 10 cm depth using EFF feature extraction. Employing a DT-EFF combined classifier, we achieved a classification accuracy of 92.16%. As shown in the confusion matrix in Figure 3, when the number of samples in a specific category was reduced to 10, although the overall accuracy exhibited inconsistent variations, the confusion matrix revealed a significant decline in the recognition rate for that category, even dropping to zero.

This occurs because the quantity of minority class samples is substantially smaller than the total dataset size. Consequently, misclassifications of minority class samples contribute less to the overall error, diminishing their influence on the aggregate accuracy metric. However, if we focus solely on the overall accuracy, this critical degradation in minority class performance may be overlooked. This phenomenon is highly detrimental to the assessment of soil pesticide residues, as it can significantly impair the classification accuracy of minority pesticide classes within the soil.

### 4.2. Effects of Pesticide Varieties on Oversampling Performance

SMOTE and G-SMOTE were applied to the training folds of the randomly selected minority classes. Table 5 reports pesticide-specific minority-class recognition rates under the KNN-EFF framework at 10 cm depth. The results indicate that oversampling improved the recognition of some minority classes, although the magnitude of improvement differed among pesticide categories.

The observed changes in oversampling performance differed among pesticides and feature representations. Although the commercial pesticide formulations showed different GC-MS volatile profiles, the present study did not directly test the relationship among volatile composition, sensor-response morphology, and class-specific model performance. Therefore, the chemical interpretation should be regarded as hypothesis-generating rather than mechanistic evidence. Future work should combine representative sensor-response curves with multivariate visualization and formal association analysis.

Although several model combinations achieved very high classification accuracy, these results should be interpreted cautiously. The samples were prepared and measured under controlled laboratory conditions, and the dataset was obtained from a limited range of soil sources, pesticide types, and experimental settings. Therefore, the high accuracy values do not necessarily indicate equivalent performance under complex field conditions. The use of training-fold-only oversampling reduced the risk of data leakage, but further validation with independent external datasets is still necessary.

In addition, although ten-fold cross-validation was used to reduce dependence on a single training/test split, the present study did not include repeated random cross-validation with multiple random seeds. Therefore, the reported accuracies do not include standard deviations across repeated independent partitions. This limitation is particularly relevant for the imbalanced-data experiments because both the random selection of minority samples and the synthetic sample generation in SMOTE/G-SMOTE may introduce additional variability. Future studies should repeat the cross-validation procedure several times with different random seeds and report the results as mean ± standard deviation. Independent external validation using field-contaminated soil samples should also be performed to further assess model robustness and generalization.

### 4.3. Comparative Analysis of Feature Extraction Methods with Data Augmentation

**Figure 4:**
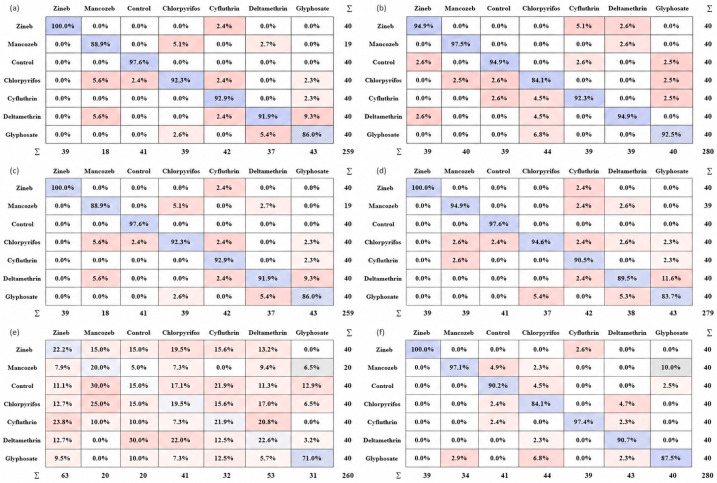
Confusion matrices for mancozeb classification at 20 cm depth under different feature extraction and data augmentation schemes: (**a**) wavelet transform (WT) baseline; (**b**) WT with SMOTE processing; (**c**) interval value (IV) baseline; (**d**) IV with SMOTE processing; (**e**) Gaussian fitting feature (GFF) baseline; (**f**) GFF with SMOTE processing. Blue cells on the main diagonal represent correctly classified samples, whereas red cells in the off-diagonal positions represent misclassified samples. A darker color indicates a higher classification percentage.

To investigate the effects of oversampling under different feature extraction methods, data for mancozeb at 20 cm depth under three feature extraction techniques—WT, IV, and GFF—were selected. Twenty data points were randomly removed to artificially create an imbalanced dataset. Two oversampling algorithms were then applied to the minority class. Table 6 shows the average accuracy before and after oversampling under different feature extraction methods, and Figure 4 presents the corresponding confusion matrices under the decision tree (DT) classifier to further examine minority-class recognition performance after SMOTE-based oversampling.

The feature extraction method directly influences the quality of the original data representation, thereby determining the effectiveness of the synthetic samples generated by oversampling algorithms in the feature space. As shown in Table 6, the improvement effects after SMOTE processing vary across different feature extraction methods in the mancozeb dataset at 20 cm depth. Among them, the average accuracy increased from 95.52% to 95.93% under GFF features and from 96.31% to 96.67% under IV features, whereas a slight decrease was observed under WT features, from 97.21% to 96.13%. In addition to the average accuracy shown in Table 6, the minority-class recall of mancozeb was directly examined from the confusion matrices in Figure 4. The results showed that SMOTE changed the mancozeb minority-class recall from 88.9% to 97.5% under WT features, from 88.9% to 94.9% under IV features, and from 20.0% to 97.1% under GFF features. These results indicate that changes in average accuracy may not fully reflect the improvement in minority-class recognition. In particular, although the average accuracy under GFF features increased only slightly from 95.52% to 95.93%, the minority-class recall of mancozeb increased markedly from 20.0% to 97.1%, demonstrating the necessity of reporting class-specific metrics in addition to average accuracy.

These results indicate that the effect of oversampling depends strongly on feature representation. SMOTE improved the average accuracy under IV and GFF features but reduced performance under WT features, while G-SMOTE did not consistently improve the results in this setting. Therefore, oversampling should be regarded as feature- and classifier-dependent rather than universally beneficial, and its effectiveness should be evaluated jointly with class-specific recall.

## 5. Conclusions

This study proposed a chamber–sensor-array–algorithm framework for volatile-fingerprint classification of pesticide-treated loess soil. A mirror-symmetric conformal gas-sensing chamber was designed and optimized using CFD simulation and orthogonal experiments, providing a more uniform gas-delivery basis for a 26-sensor MOS array.

At the algorithmic level, eight feature extraction methods, four classifiers, and two oversampling algorithms were evaluated. Under controlled laboratory cross-validation, several classifier–feature combinations achieved high apparent classification accuracy, and SMOTE improved minority-class recognition in selected pesticide–depth–feature combinations. However, the high accuracies, including values close to or equal to 100%, should be interpreted cautiously because the dataset was limited in sample size, soil-source diversity, pesticide formulations, and environmental variability.

Overall, this work contributes a rapid electronic-nose-based volatile-fingerprint classification strategy for pesticide-treated soil and highlights the importance of class-specific metrics for imbalanced pesticide datasets. Future work should include quantitative chemical validation, larger independent field datasets, controlled interference tests, and repeated validation with statistical uncertainty.

## Figures and Tables

**Table 1:** Single-factor level assignment table.

Factors	Code	Level				
		1	2	3	4	5
Transition region width (mm)	P	2	2.5	3	3.5	4
Inlet flow rate (L/min)	Q	1.1	1.3	1.5	1.7	1.9
Conformal circle diameter (mm)	L	9	10	11	12	13
Flow guide cavity thickness (mm)	R	16	18	20	22	24

**Table 2:** Response characteristics of sensor array.

No.	Sensor Model	Target Gas	Sensitivity (Resistance Change Ratio)
S1	TGS2600	Ethanol, hydrogen	0.30~0.60 Rs (in 10 ppm hydrogen)/Rs (in air)
S2	TGS2602	Hydrogen sulfide, ammonia	0.08~0.50 Rs (in 10 ppm ethanol)/Rs (in air)
S3	TGS2603	Air pollutants	<0.50 Rs (in 10 ppm ethanol)/Rs (in air)
S4	TGS2610	Liquefied petroleum gas	0.56 ± 0.06 Rs (3000 ppm)/Rs (1000 ppm)
S5	TGS2611	Methane	0.56 ± 0.06 Rs (9000 ppm)/Rs (3000 ppm)
S6	TGS2612	Methane, propane, butane mixture	0.50~0.65 Rs (9000 ppm)/Rs (3000 ppm)
S7	TGS2618	LPG-butane composite	0.56 ± 0.06 Rs (3000 ppm)/Rs (1000 ppm)
S8	TGS2620	Ethanol, organic solvents	0.30~0.50 Rs (in 300 ppm ethanol)/Rs (in 50 ppm ethanol)
S9	GSBT11	Toluene/formaldehyde/benzene mixture	0.50~0.70 Rs (in 5 ppm toluene)/Rs (in air)
S10	MS1100	VOCs, organic compounds & smoke	0.40~0.60 Rs (in 5 ppm toluene)/Rs (in air)
S11	MP-2	Propane with air pollutants	≥3 Rs (in air)/Rs (in 2000 ppm propane)
S12	MP-4	Methane in natural gas/biogas	≥5 Rs (in air)/Rs (in 5000 ppm methane)
S13	MP-5	Liquefied petroleum gas	≥5 Rs (in air)/Rs (in 2000 ppm propane)
S14	MP-7	Carbon monoxide	≥3 Rs (in air)/Rs (in 100 ppm carbon monoxide)
S15	MP-9	Carbon monoxide–methane mixture	≥3 Rs (in air)/Rs (in 100 ppm carbon monoxide)
S16	MP-3B	Ethanol	≥3 Rs (in air)/Rs (in 50 ppm alcohol)
S17	MP135	$H_{2}$/ethanol/CO blend	≥3 Rs (in air)/Rs (in 50 ppm H_2_)
S18	MP402	Methane in natural gas/biogas	≥5 Rs (in air)/Rs (in 5000 ppm methane)
S19	MP503	Ethanol–smoke–butane–formaldehyde mixture	≥5 Rs (in air)/Rs (in 50 ppm alcohol)
S20	MP702	Ammonia	≥5 Rs (in air)/Rs (in 50 ppm ammonia)
S21	MP801	BTEX–formaldehyde–ethanol	≥2 Rs (in air)/Rs (in 10 ppm alcohol)
S22	MP901	Benzene/acetone/paint solvents	≥5 Rs (in air)/Rs (in 10 ppm alcohol)
S23	MP905	BTEX–formaldehyde–ethanol–smoke	≥2 Rs (in air)/Rs (in 10 ppm alcohol)
S24	WSP1110	Nitrogen dioxide	≤0.1 Rs (in air)/Rs (in 2 ppm nitrogen dioxide)
S25	WSP2110	BTEX–formaldehyde–ethanol–acetone	≥3 Rs (in air)/Rs (in 10 ppm toluene)
S26	WSP7110	$H_{2}S$–acetylene–benzene mixture	≥5 Rs (in air)/Rs (in 50 ppm hydrogen sulfide)

**Table 3:** Gas chromatography–mass spectrometry determination results.

	Glyphosate	Chlorpyrifos	Cyfluthrin	Deltamethrin	Mancozeb	Zineb
Other	3	1	0	1	3	3
Ester	2	0	0	0	0	6
Amide	0	0	1	0	0	0
Alkene	0	0	0	0	0	3
Alkane	8	0	0	0	11	10
Ketone	0	0	0	0	1	2
Aldehyde	1	0	0	0	3	1
Ether	2	0	0	0	0	2
Nitrile	0	0	0	0	0	1
Halohydrocarbon	0	1	1	0	0	0
Phenol	0	0	0	1	0	0
Aromatic hydrocarbon	2	23	35	18	3	8
Alcohol	10	0	0	1	3	4

**Table 4:** Average classification accuracy of pesticide volatile-fingerprint classes at different soil depths.

Depth	Classifier	MAX	Mean	PFF	IV	WT	EFF	GFF	FT
10 cm	KNN	100.00	100.00	100.00	100.00	100.00	61.47	61.92	100.00
10 cm	DT	99.75	98.09	96.28	98.10	96.81	92.16	96.56	96.63
10 cm	SVM	100.00	99.88	99.08	99.90	99.89	84.25	89.18	99.14
10 cm	NN	100.00	99.88	98.95	99.98	99.99	80.00	85.34	99.63
20 cm	KNN	100.00	100.00	100.00	100.00	100.00	60.23	61.33	100.00
20 cm	DT	95.52	96.85	96.25	96.85	96.49	92.47	95.48	96.84
20 cm	SVM	100.00	100.00	99.55	100.00	99.99	86.47	87.33	99.38
20 cm	NN	100.00	100.00	99.74	100.00	100.00	81.48	84.25	99.74
30 cm	KNN	100.00	100.00	99.99	100.00	100.00	59.85	58.33	99.99
30 cm	DT	97.77	95.84	96.68	96.04	96.23	87.62	95.92	96.24
30 cm	SVM	99.91	99.76	98.83	99.80	99.48	79.56	86.60	98.87
30 cm	NN	100.00	99.99	99.37	99.98	99.95	77.98	85.15	99.73
40 cm	KNN	100.00	100.00	99.99	100.00	100.00	59.85	58.33	99.99
40 cm	DT	97.77	98.76	98.66	98.29	97.39	78.78	96.45	97.22
40 cm	SVM	99.91	96.67	98.83	98.05	99.89	82.61	89.42	99.67
40 cm	NN	100.00	99.99	98.73	98.98	99.59	79.34	89.23	99.99

**Table 5:** Minority-class recognition rate before and after oversampling treatment.

	Glyphosate	Deltamethrin	Cyfluthrin
Unbalanced	89.82%	94.51%	91.12%
SMOTE	95.65%	95.17%	92.33%
G-SMOTE	90.41%	96.12%	91.36%

**Table 6:** Average accuracy before and after oversampling across different feature extraction methods.

	WT	IV	GFF
Unbalanced	97.21%	96.31%	95.52%
SMOTE	96.13%	96.67%	95.93%
G-SMOTE	90.16%	92.44%	93.64%

## Data Availability

The data presented in this study are available on reasonable request from the corresponding author. The data are not publicly available due to privacy or ethical restrictions.

## Supplementary Materials

The following supporting information can be downloaded at: https://www.mdpi.com/article/10.3390/s26154763/s1, Table S1. Classification and nominal active-ingredient contents of the commercial pesticide formulations used in the soil-leaching experiments. Table S2. Volatile compounds identified in the six commercial pesticide formulations by headspace solid-phase microextraction coupled with gas chromatography–mass spectrometry.

## References

[1] Frazão L.A., Santana I.K.D.S., Campos D.V.B.D., Feigl B.J., Cerri C.C. (2010). Carbon and nitrogen stocks and organic matter light fraction in a Typic Quartzipsamment under agricultural use. Braz. Agric. Res..

[2] Word M.L., Hall S.J., Robinson B.E., Manneh B., Beye A., Cease A.J. (2019). Soil-targeted interventions could alleviate locust and grasshopper pest pressure in West Africa. Sci. Total Environ..

[3] Wan N.-F., Fu L., Dainese M., Kiær L.P., Hu Y.-Q., Xin F., Goulson D., Woodcock B.A., Vanbergen A.J., Spurgeon D.J. (2025). Pesticides have negative effects on non-target organisms. Nat. Commun..

[4] Punniyakotti P., Vinayagam S., Rajamohan R., Priya S., Moovendhan M., Sundaram T. (2024). Environmental fate and ecotoxicological behaviour of pesticides and insecticides in non-target environments: Nanotechnology-based mitigation strategies. J. Environ. Chem. Eng..

[5] Vasilevskaya L.N., Lisina I.A., Semiletov I.P., Stochkute J.V., Vasilevskii D.N. (2025). Influence of Meteorological Factors on the Seasonal Runoff of the Kolyma River. Russ. Meteorol. Hydrol..

[6] Tran H.N., Nguyen N.B., Ly N.H., Joo S.-W., Vasseghian Y. (2023). Core-shell Au@ ZIF-67-based pollutant monitoring of thiram and carbendazim pesticides. Environ. Pollut..

[7] Chao K., Schmidt W., Qin J., Kim M. (2022). A rapid and precise spectroscopic method for detecting fipronil insecticide on solid surfaces. J. Food Meas. Charact..

[8] Nazarloo A.S., Sharabiani V.R., Gilandeh Y.A., Taghinezhad E., Szymanek M. (2021). Evaluation of different models for non-destructive detection of tomato pesticide residues based on near-infrared spectroscopy. Sensors.

[9] Lin K., Wang Z., Wang L., Zhou J., Han L., Wen J., Dong T., Duan R., Li N. (2023). A Rapid Determination of 30 Prohibited Pesticide Residues in Lycii Fructus by UPLC-MS/MS. Phyton.

[10] Martínez Vidal J.L., Padilla Sánchez J.A., Plaza-Bolaños P., Garrido Frenich A., Romero-González R. (2010). Use of pressurized liquid extraction for the simultaneous analysis of 28 polar and 94 non-polar pesticides in agricultural soils by GC/QqQ-MS/MS and UPLC/QqQ-MS/MS. J. AOAC Int..

[11] Delattre F., Cazier F., Cazier F., Tine A. (2009). Use a fluorescent molecular sensor for the detection of pesticides and herbicides in water. Curr. Anal. Chem..

[12] Chowdhury N.M., Soliman M., Alenezi A.M., Alam T., Alsaif H., Soliman M.S., Islam M.T. (2024). Sensitivity detection of imidacloprid pesticide using a metasurface sensor in THz spectrum regime. Eng. Sci. Technol. An. Int. J..

[13] De Stefano L., Moretti L., Rendina I., Rotiroti L. (2005). Pesticides detection in water and humic solutions using porous silicon technology. Sens. Actuators B Chem..

[14] Shi T., Zhang Q., Chen X., Mao G., Feng W., Yang L., Zhao T., Wu X., Chen Y. (2024). Overview of deltamethrin residues and toxic effects in the global environment. Environ. Geochem. Health.

[15] Singh R., Shukla A., Kaur G., Girdhar M., Malik T., Mohan A. (2024). Systemic analysis of glyphosate impact on environment and human health. ACS Omega.

[16] van Wijngaarden R.P.A., Brink P.J.V.D., Crum S.J.H., Brock T.C.M., Leeuwangh P., Voshaar O.J.H. (1996). Effects of the insecticide Dursban^®^ 4E (active ingredient chlorpyrifos) in outdoor experimental ditches: I. Comparison of short-term toxicity between the laboratory and the field. Environ. Toxicol. Chem..

[17] Sharma A., Katna S., Dubey J.K., Sharma S., Istatu P.S., Devi N., Brar G.S., Kumar A., Singh S., Prashad H. (2024). Residue behaviour and health risk assessment of chlorpyrifos and mancozeb in apple fruits and soil. Environ. Monit. Assess..

[18] (2014). Test Guidelines on Environmental Safety Assessment for Chemical Pesticides—Part 5: Soil Leaching Test.

[19] Zheng Z., Zhang C. (2022). Electronic noses based on metal oxide semiconductor sensors for detecting crop diseases and insect pests. Comput. Electron. Agric..

[20] Nakata S., Kashima K. (2012). Distinction between alcohols and hydrocarbons with a semiconductor gas sensor depending on the range and frequency of a cyclic temperature. Anal. Methods.

[21] Lu L., Hu Z., Hu X., Li D., Tian S. (2022). Electronic tongue and electronic nose for food quality and safety. Food Res. Int..

[22] Cai M., Xu S., Zhou X., Lu H. (2024). Electronic nose humidity compensation system based on rapid detection. Sensors.

[23] Borah K., Das H.S., Seth S., Mallick K., Rahaman Z., Mallik S. (2024). A review on advancements in feature selection and feature extraction for high-dimensional NGS data analysis. Funct. Integr. Genom..

[24] Yang W., Qin Y., Huang R., Chen Y. (2025). Adaptive feature extraction for entity relation extraction. Comput. Speech Lang..

[25] Daßler N., Günther T. (2024). Variational feature extraction in scientific visualization. ACM Trans. Graph. (TOG).

[26] Wang C.X., Dong L.L., Pan Z.M., Zhang T. (2012). Classification for unbalanced dataset by an improved KNN algorithm based on weight. Int. Inf. Inst. Inf..

[27] Zhu X., Wan W., Qian L., Cai Y., Chen X., Zhang P., Huang G., Liu B., Yao Q., Li S. (2023). Research on intelligent identification and grading of nonmetallic inclusions in steels based on deep learning. Micromachines.

[28] Douzas G., Bacao F. (2019). Geometric SMOTE a geometrically enhanced drop-in replacement for SMOTE. Inf. Sci..

[29] Chen H., Huo D., Zhang J. (2022). Gas recognition in E-nose system: A review. IEEE Trans. Biomed. Circuits Syst..

[30] Cheng K., Zhang C., Yu H., Yang X., Zou H., Gao S. (2019). Grouped SMOTE with noise filtering mechanism for classifying imbalanced data. IEEE Access.

[31] Zhang C., Chen M., Zhang Y., Deng W., Gong Y., Zhang D. (2024). Partial discharge pattern recognition algorithm of overhead covered conductors based on feature optimization and bidirectional LSTM-GRU. IET Gener. Transm. Distrib..

[32] Acharya S., Kar T., Samal U.C., Patra P.K. (2023). Performance comparison between svm and ls-svm for rice leaf disease detection. EAI Endorsed Trans. Scalable Inf. Syst..

[33] Abolhosseini S., Khorashadizadeh M., Chahkandi M., Golalizadeh M. (2024). A modified ID3 decision tree algorithm based on cumulative residual entropy. Expert Syst. Appl..

[34] Ukey N., Yang Z., Li B., Zhang G., Hu Y., Zhang W. (2023). Survey on exact knn queries over high-dimensional data space. Sensors.

[35] Dou H., Shen F., Zhao J., Mu X. (2023). Understanding neural network through neuron level visualization. Neural Netw..

