# Supplementary material for: Sensor Array and SMOTE-Based Algorithms for Volatile-Fingerprint Classification of Pesticide-Treated Soil with a Novel Chamber
Source: Sensors (Basel). 2026 Jul 27;26(15):4763. doi: 10.3390/s26154763 (PMC13469941; doi:10.3390/s26154763)
Supplement: Supplementary file 1 [file sensors-26-04763-s001.zip › sensors-4438455-supplementary.pdf]

Table S1. Classification and nominal active-ingredient contents of the commercial pesticide formulations used in the soil-leaching experiments.

Pesticide class	Pesticide	Intended use	Nominal active-ingredient content (%)
Pyrethroid	Deltamethrin	Insecticide	2.8
Pyrethroid	Cyfluthrin	Insecticide	5.7
Organophosphorus compound	Glyphosate	Herbicide	41
Organophosphorus compound	Chlorpyrifos	Insecticide	5
Organosulfur/dithiocarbamate	Zineb	Fungicide	80
Organosulfur/dithiocarbamate	Mancozeb	Fungicide	80

Table S2. Volatile compounds identified in the six commercial pesticide formulations by headspace solid-phase microextraction coupled with gas chromatography–mass spectrometry.

Pesticide	CAS No.	Compound name	Formula	Chemical class
Glyphosate	1004-29-1	2-Butyltetrahydrofuran	C8H16O	Other
Glyphosate	10042-59-8	1-Decanol	C10H22O	Alcohol
Glyphosate	111-87-5	1-Octanol	C8H18O	Alcohol
Glyphosate	1119-87-5	Aliphatic diol	C12H26O2	Alcohol
Glyphosate	112-30-1	1-Decanol	C10H22O	Alcohol
Glyphosate	112-31-2	Decanal	C10H20O	Aldehyde
Glyphosate	112-53-8	1-Dodecanol	C12H26O	Alcohol
Glyphosate	1120-21-4	Undecane	C11H24	Alkane
Glyphosate	1560-95-8	2-Methyltetradecane	C15H32	Alkane
Glyphosate	1653-30-1	2-Undecanol	C11H24O	Alcohol
Glyphosate	1724-39-6	Cyclododecanol	C12H24O	Alcohol
Glyphosate	18435-22-8	3-Methyltetradecane	C15H32	Alkane
Glyphosate	18675-24-6	1-Decanol, 2-methyl-	C11H24O	Alcohol
Glyphosate	213406-04-3	Decyl pentyl ether	C15H32O	Ether
Glyphosate	22972-35-6	Spiro[fluorene]	C13H16	Aromatic hydrocarbon
Glyphosate	3055-93-4	Diethylene glycol monodecyl ether	C16H34O3	Alcohol
Glyphosate	40853-55-2	5-Methyl-2-isopropyl-1-hexanol acetate	C12H24O2	Ester
Glyphosate	475-03-6	Ionene	C13H18	Aromatic hydrocarbon
Glyphosate	50789-31-6	5,11-Diethyl-8-methyl-7,9-dioxapentadecane	C18H38O2	Other
Glyphosate	544-76-3	Hexadecane	C16H34	Alkane
Glyphosate	629-50-5	Tridecane	C13H28	Alkane
Glyphosate	629-59-4	Tetradecane	C14H30	Alkane
Glyphosate	629-62-9	Pentadecane	C15H32	Alkane
Glyphosate	629-78-7	Heptadecane	C17H36	Alkane

Pesticide	CAS No.	Compound name	Formula	Chemical class
Glyphosate	629-82-3	Octyl ether	C16H34O	Ether
Glyphosate	79059-78-2	Decyl formate	C11H22O2	Ester
Glyphosate	85763-57-1	11-Methyldodecanol	C13H28O	Alcohol
Glyphosate	931-35-1	2-Ethyl-4,5-dihydro-4-methylimidazole	C6H12N2	Other
Chlorpyrifos	100-41-4	Ethylbenzene	C8H10	Aromatic hydrocarbon
Chlorpyrifos	1074-17-5	Isopropyltoluene	C10H14	Aromatic hydrocarbon
Chlorpyrifos	108-90-7	Chlorobenzene	C6H5Cl	Halohydrocarbon
Chlorpyrifos	135-98-8	1-Methylpropylbenzene	C10H14	Aromatic hydrocarbon
Chlorpyrifos	13632-94-5	1,4-Diethyl-2-methylbenzene	C11H16	Aromatic hydrocarbon
Chlorpyrifos	1587-04-8	Isopentenyltoluene	C10H12	Aromatic hydrocarbon
Chlorpyrifos	2050-24-0	1,3-Diethyl-5-methylbenzene	C11H16	Aromatic hydrocarbon
Chlorpyrifos	275-51-4	Azulene	C10H8	Aromatic hydrocarbon
Chlorpyrifos	2921-88-2	Cypermethrin	C9H11Cl3NO3PS	Other
Chlorpyrifos	3145-76-4	1-Methyl-2-phenylcyclopropane	C10H12	Aromatic hydrocarbon
Chlorpyrifos	4132-77-8	3,4-Dimethyl-tert-butylbenzene	C11H16	Aromatic hydrocarbon
Chlorpyrifos	4218-48-8	1-Ethyl-4-isopropylbenzene	C11H16	Aromatic hydrocarbon
Chlorpyrifos	496-11-7	Indane	C9H10	Aromatic hydrocarbon
Chlorpyrifos	526-73-8	1,2,3-Trimethylbenzene	C9H12	Aromatic hydrocarbon
Chlorpyrifos	538-93-2	2-Methylpropylbenzene	C10H14	Aromatic hydrocarbon
Chlorpyrifos	611-14-3	1-Ethyl-2-methylbenzene	C9H12	Aromatic hydrocarbon
Chlorpyrifos	613-33-2	4,4'-Dimethylbiphenyl	C14H14	Aromatic hydrocarbon
Chlorpyrifos	622-96-8	1-Methyl-4-ethylbenzene	C9H12	Aromatic hydrocarbon
Chlorpyrifos	767-58-8	1-Methylindene	C10H12	Aromatic hydrocarbon
Chlorpyrifos	87-85-4	Hexamethylbenzene	C12H18	Aromatic hydrocarbon

Pesticide	CAS No.	Compound name	Formula	Chemical class
Chlorpyrifos	874-41-9	1-Ethyl-2,4-dimethylbenzene	C10H14	Aromatic hydrocarbon
Chlorpyrifos	91-57-6	2-Methylnaphthalene	C11H10	Aromatic hydrocarbon
Chlorpyrifos	939-27-5	2-Ethylnaphthalene	C12H12	Aromatic hydrocarbon
Chlorpyrifos	97664-19-2	1-Methyl-2-(1-methyl-2-propenyl)benzene	C11H14	Aromatic hydrocarbon
Chlorpyrifos	99-87-6	p-Xylene	C10H14	Aromatic hydrocarbon
Cyfluthrin	100-41-4	Ethylbenzene	C8H10	Aromatic hydrocarbon
Cyfluthrin	105-05-5	1,4-Diethylbenzene	C10H14	Aromatic hydrocarbon
Cyfluthrin	106-42-3	p-Xylene	C8H10	Aromatic hydrocarbon
Cyfluthrin	1074-17-5	1-Methyl-2-propylbenzene	C10H14	Aromatic hydrocarbon
Cyfluthrin	108-90-7	Chlorobenzene	C6H5Cl	Halohydrocarbon
Cyfluthrin	135-98-8	1-Methylpropylbenzene	C10H14	Aromatic hydrocarbon
Cyfluthrin	13632-94-5	1,4-Diethyl-2-methylbenzene	C11H16	Aromatic hydrocarbon
Cyfluthrin	1587-04-8	1-Methyl-2-(2-propenyl)benzene	C10H12	Aromatic hydrocarbon
Cyfluthrin	19692-45-6	1-(Chloromethyl)-4-(1,1-dimethylethyl)benzene	C11H15Cl	Halohydrocarbon
Cyfluthrin	2050-24-0	1,3-Diethyl-5-methylbenzene	C11H16	Aromatic hydrocarbon
Cyfluthrin	2131-41-1	1,4,5-Trimethylnaphthalene	C13H14	Aromatic hydrocarbon
Cyfluthrin	21693-54-9	5,7-Dimethyltetralin	C13H18	Aromatic hydrocarbon
Cyfluthrin	2245-38-7	1,6,7-Trimethylnaphthalene	C13H14	Aromatic hydrocarbon
Cyfluthrin	275-51-4	Azulene	C10H8	Aromatic hydrocarbon
Cyfluthrin	2921-88-2	Cypermethrin	C9H11Cl3NO3PS	Other
Cyfluthrin	30316-17-7	2,5,8-Trimethyltetralin	C13H18	Aromatic hydrocarbon
Cyfluthrin	3145-76-4	1-Methyl-2-phenylcyclopropane	C10H12	Aromatic hydrocarbon
Cyfluthrin	4132-77-8	3,4-Dimethylisopropylbenzene	C11H16	Aromatic hydrocarbon
Cyfluthrin	4810-04-2	1,3,5-Trimethyl-2-propylbenzene	C12H18	Aromatic hydrocarbon

Pesticide	CAS No.	Compound name	Formula	Chemical class
Cyfluthrin	4957-14-6	Di-p-tolylmethane	C15H16	Aromatic hydrocarbon
Cyfluthrin	496-11-7	Indane	C9H10	Aromatic hydrocarbon
Cyfluthrin	527-84-4	o-Trimethylbenzene	C10H14	Aromatic hydrocarbon
Cyfluthrin	575-41-7	1,3-Dimethylnaphthalene	C12H12	Aromatic hydrocarbon
Cyfluthrin	611-14-3	1-Ethyl-2-methylbenzene	C9H12	Aromatic hydrocarbon
Cyfluthrin	61141-66-0	3,4-Diethylbiphenyl	C16H18	Aromatic hydrocarbon
Cyfluthrin	612-00-0	1,1-Diphenylethane	C14H14	Aromatic hydrocarbon
Cyfluthrin	613-33-2	4,4'-Dimethylbiphenyl	C14H14	Aromatic hydrocarbon
Cyfluthrin	622-96-8	1-Ethyl-4-methylbenzene	C8H10	Aromatic hydrocarbon
Cyfluthrin	643-93-6	3-Methylbiphenyl	C13H12	Aromatic hydrocarbon
Cyfluthrin	6682-71-9	4,7-Dimethylindane	C11H14	Aromatic hydrocarbon
Cyfluthrin	713-36-0	o-Benzyltoluene	C14H14	Aromatic hydrocarbon
Cyfluthrin	87-85-4	Hexamethylbenzene	C12H18	Aromatic hydrocarbon
Cyfluthrin	877-44-1	1,2,4-Triethylbenzene	C12H18	Aromatic hydrocarbon
Cyfluthrin	91-57-6	2-Methylnaphthalene	C11H10	Aromatic hydrocarbon
Cyfluthrin	934-80-5	4-Ethyl-1,2-dimethylbenzene	C10H14	Aromatic hydrocarbon
Cyfluthrin	939-27-5	2-Ethylnaphthalene	C12H12	Aromatic hydrocarbon
Cyfluthrin	95-93-2	1,2,4,5-Tetramethylbenzene	C10H14	Aromatic hydrocarbon
Cyfluthrin	97664-18-1	1-Methyl-4-(1-methyl-2-propenyl)benzene	C11H14	Aromatic hydrocarbon
Deltamethrin	104-76-7	2-Ethylhexanol	C8H18O	Alcohol
Deltamethrin	105-05-5	p-Diethylbenzene	C10H14	Aromatic hydrocarbon
Deltamethrin	1074-43-7	m-Methylpropylbenzene	C10H14	Aromatic hydrocarbon
Deltamethrin	1074-55-1	p-Methylpropylbenzene	C10H14	Aromatic hydrocarbon
Deltamethrin	128-37-0	2,6-Di-tert-butyl-4-methylphenol	C15H24O	Phenol

Pesticide	CAS No.	Compound name	Formula	Chemical class
Deltamet hrin	135-01-3	o-Diethylbenzene	C10H14	Aromatic hydrocarbon
Deltamet hrin	13632-94-5	1-Methyl-2,5-diethylbenzene	C11H16	Aromatic hydrocarbon
Deltamet hrin	1595-16-0	2-p-Tolylbutane	C11H16	Aromatic hydrocarbon
Deltamet hrin	2050-24-0	1-Methyl-3,5-diethylbenzene	C11H16	Aromatic hydrocarbon
Deltamet hrin	2719-52-0	(2-Pentyl)benzene	C11H16	Aromatic hydrocarbon
Deltamet hrin	275-51-4	Azulene	C10H8	Aromatic hydrocarbon
Deltamet hrin	3145-76-4	1-Methyl-2-phenylcyclopropane	C10H12	Aromatic hydrocarbon
Deltamet hrin	4132-77-8	3,4-Dimethylheptene	C11H16	Aromatic hydrocarbon
Deltamet hrin	527-84-4	o-Methylstyrene	C10H14	Aromatic hydrocarbon
Deltamet hrin	611-14-3	o-Ethyltoluene	C9H12	Aromatic hydrocarbon
Deltamet hrin	622-96-8	4-Ethyltoluene	C9H12	Aromatic hydrocarbon
Deltamet hrin	767-58-8	1-Methylindene	C10H12	Aromatic hydrocarbon
Deltamet hrin	791-28-6	Triphenylphosphine oxide	C18H15OP	Other
Deltamet hrin	934-80-5	1-Ethyl-3,4-dimethylbenzene	C10H14	Aromatic hydrocarbon
Deltamet hrin	939-27-5	2-Ethylnaphthalene	C12H12	Aromatic hydrocarbon
Deltamet hrin	95-93-2	1,2,4,5-Tetramethylbenzene	C10H14	Aromatic hydrocarbon
Mancozeb	100-41-4	Ethylbenzene	C8H10	Aromatic hydrocarbon
Mancozeb	1002-43-3	3-Methyldecane	C12H26	Alkane
Mancozeb	106-42-3	p-Xylene	C8H10	Aromatic hydrocarbon
Mancozeb	108-38-3	m-Xylene	C8H10	Aromatic hydrocarbon
Mancozeb	111-65-9	Octane	C8H18	Alkane
Mancozeb	112-30-1	1-Decanol	C10H22O	Alcohol
Mancozeb	112-31-2	Decanal	C10H20O	Aldehyde
Mancozeb	112-40-3	Dodecane	C12H26	Alkane
Mancozeb	1192-58-1	2-Formyl-1-methylpyrrole	C6H7NO	Aldehyde
Mancozeb	124-18-5	Decane	C10H22	Alkane
Mancozeb	124-19-6	Nonanal	C9H18O	Aldehyde
Mancozeb	13150-81-7	2,6-Dimethyldecane	C12H26	Alkane

Pesticide	CAS No.	Compound name	Formula	Chemical class
Mancozeb	13475-78-0	2-Methyl-5-ethylheptane	C10H22	Alkane
Mancozeb	13475-82-6	Marukazol R	C12H26	Alkane
Mancozeb	2001-93-6	2,4(1H,3H)-Pyrimidinedithione	C4H4N2S2	Other
Mancozeb	21078-65-9	2-Ethyldecanol	C12H26O	Alcohol
Mancozeb	25870-62-6	1-Phenyl-2-hexanone	C12H16O	Ketone
Mancozeb	289-16-7	1,2,4-Trithiolane	C2H4S3	Other
Mancozeb	556-61-6	Tropex	C2H3NS	Other
Mancozeb	5911-04-6	(±)-3-Methylnonane	C10H22	Alkane
Mancozeb	62108-24-1	Decane, 2,6,6-trimethyl-	C13H28	Alkane
Mancozeb	62183-79-3	2,2,4,4-Tetramethyloctane	C12H26	Alkane
Mancozeb	629-59-4	Tetradecane	C14H30	Alkane
Mancozeb	931-17-9	1,2-Cyclohexanediol	C6H12O2	Alcohol
Zineb	100-52-7	Benzaldehyde	C7H6O	Aldehyde
Zineb	1004-29-1	Furan, 2-butyltetrahydro-	C8H16O	Other
Zineb	10099-70-4	Diisopropyl maleate	C10H16O4	Ester
Zineb	10374-74-0	7-Tetradecene	C14H28	Alkene
Zineb	112-40-3	Dodecane	C12H26	Alkane
Zineb	112-53-8	1-Dodecanol	C12H26O	Alcohol
Zineb	112-66-3	Laurate ester	C14H28O2	Ester
Zineb	112-72-1	1-Tetradecanol	C14H30O	Alcohol
Zineb	1120-21-4	Undecane	C11H24	Alkane
Zineb	1196-01-6	l-Menthone	C10H14O	Ketone
Zineb	124-18-5	Decane	C10H22	Alkane
Zineb	1792-81-0	cis-1,2-Cyclohexanediol	C6H12O2	Alcohol
Zineb	18335-15-4	(1-Ethylhexyl)benzene	C14H22	Aromatic hydrocarbon
Zineb	1953-99-7	1,2-Benzenedicarbonitrile, 3,4,5,6-tetrachloro-	C8Cl4N2	Nitrile
Zineb	1985-57-5	(1,1-Dimethylbutyl)benzene	C12H18	Aromatic hydrocarbon
Zineb	2049-95-8	(1,1-Dimethylpropyl)benzene	C11H16	Aromatic hydrocarbon
Zineb	2132-84-5	(1-Methylhexyl)benzene	C13H20	Aromatic hydrocarbon
Zineb	2416945-47-4	Heterocyclopentanoic acid, heptyl ester	C16H24O2	Ester
Zineb	2719-52-0	(1-Methylbutyl)benzene	C11H16	Aromatic hydrocarbon
Zineb	289-16-7	1,2,4-Trithiolane	C2H4S3	Other
Zineb	29138-94-	Pentadecane,	C22H38	Aromatic

Pesticide	CAS No.	Compound name	Formula	Chemical class
	1	2-methyl-2-phenyl-benzene		hydrocarbon
Zineb	3482-63-1	Dodecane, 1-methoxy-	$C_{13}H_{28}O$	Ether
Zineb	4170-84-7	(1,1-Diethylpropyl)benzene	$C_{13}H_{20}$	Aromatic hydrocarbon
Zineb	544-76-3	Hexadecane	$C_{16}H_{34}$	Alkane
Zineb	556-61-6	Methane, isothiocyanato-	C_2H_3NS	Other
Zineb	593-45-3	Octadecane	$C_{18}H_{38}$	Alkane
Zineb	629-50-5	Tridecane	$C_{13}H_{28}$	Alkane
Zineb	629-59-4	Tetradecane	$C_{14}H_{30}$	Alkane
Zineb	629-62-9	Pentadecane	$C_{15}H_{32}$	Alkane
Zineb	629-78-7	Heptadecane	$C_{17}H_{36}$	Alkane
Zineb	6418-41-3	Tridecane, 3-methyl-	$C_{14}H_{30}$	Alkane
Zineb	6652-28-4	Ethanone, 2-(1-methylethoxy)-1,2-diphenyl-	$C_{17}H_{18}O_2$	Ketone
Zineb	7206-13-5	β-trans-Dodecene	$C_{12}H_{24}$	Alkene
Zineb	7206-26-0	β-cis-Dodecene	$C_{12}H_{24}$	Alkene
Zineb	7283-70-7	trans-2-Butenedioic acid, bis(1-methylethyl) ester	$C_{10}H_{16}O_4$	Ester
Zineb	7388-90-1	1-Tetradecanol, methyl ether	$C_{15}H_{32}O$	Ether
Zineb	777-22-0	(1-Methylheptyl)benzene	$C_{14}H_{22}$	Aromatic hydrocarbon
Zineb	84-69-5	1,2-Benzenedicarboxylic acid, bis(2-methylpropyl) ester	$C_{16}H_{22}O_4$	Ester
Zineb	84-74-2	Dibutyl phthalate	$C_{16}H_{22}O_4$	Ester
Zineb	98-85-1	α-Phenylethanol	$C_8H_{10}O$	Alcohol
